# Development and validation of a novel scoring scale for colonic endocytoscopy staining quality

**DOI:** 10.1038/s41598-026-37406-0

**Published:** 2026-02-04

**Authors:** Jiawei Fan, He Zhu, Mingqing Liu, Fengming Ni, Dong Yang, Guohua Jin, Ke Tao, Hong Xu

**Affiliations:** Department of Gastroenterology, The First Hospital of Jilin University, 1 Xinmin Street, Changchun, 130021 PR China

**Keywords:** Colon leision, Endocytoscopy, Methylene blue staining, Optical biopsy, Diseases, Gastroenterology, Medical research

## Abstract

Background While endocytoscopy allows for in vivo cellular-level observation, achieving high-quality staining remains a technical challenge. Aims To develop and validate a simple, reliable scale for assessing and improving endocytoscopic staining quality. Methods We developed a four-dimension staining quality score and validated it on a retrospective image cohort (n = 2692). The scale’s reliability, validity, and internal consistency were assessed, and associated factors were identified using multinomial regression. The scale was then externally validated in a separate retrospective image cohort (n = 2413) to confirm its clinical applicability. Results The scale showed inter-rater agreement (ICC = 0.992) and reliability (Cronbach’s alpha = 0.81), with a single principal factor explaining 62.3% of variance.The Spearman correlation between the gold standard score and AOD value was 0.81. Staining quality was influenced by lesion characteristics and physician experience (P < 0.05). After one month of training, junior physicians showed improved agreement with gold standard scores, with Kappa values rising from 0.61, 0.66, and 0.69 to 0.81, 0.83, and 0.82. Higher staining quality was also significantly associated with better concordance between Endocytoscopy imaging and histopathology (P < 0.001). Conclusion This endocytoscopic staining quality score is a reliable and valid tool to guide technique refinement and improve diagnostic accuracy.

**Supplementary Information:**

The online version contains supplementary material available at 10.1038/s41598-026-37406-0.

## Introduction

Endocytoscopy (EC) is an emerging ultra–high-magnification imaging technology that enables real-time optical biopsy during endoscopic examinations, facilitating visualization of cellular structures that are critical for determining the nature of identified lesions^1–4^. The primary observational focus during EC examination lies in nuclear morphology and glandular architecture within the superficial mucosal layer. Therefore, the application of appropriate staining reagents and methodologies is essential for acquiring high-quality images that allow reliable assessment^5,6^. While the incorporation of EC staining techniques represents a significant advancement in endoscopic practice, variability in staining quality frequently compromises diagnostic accuracy. This variability arises from numerous factors, including, but not limited to, the choice of staining agent, reagent concentration, staining duration, consistency of technical application, and the intrinsic characteristics of the lesion itself.

Currently, a standardized and validated scoring system for evaluating the efficacy of EC staining is lacking. Consequently, image assessment often relies heavily on the subjective experience of clinicians, resulting in considerable interobserver heterogeneity and presenting substantial challenges for novice endoscopists who lack objective benchmarks for evaluation, thereby steepening the learning curve. Beyond the staining technique per se, other factors can also adversely affect image quality. Inadequate cleansing of mucus from the mucosal surface before staining, as well as motion artifacts introduced by endoscope instability during image acquisition, can result in suboptimal visualization, thereby hindering accurate lesion characterization. Therefore, the consistent acquisition of high quality EC-stained images is paramount for fully leveraging the diagnostic potential of this technology and ensuring reliable real-time in vivo optical biopsy for lesion assessment^7^. To address this gap, the development of an assessment system that provides standardized and objective criteria for evaluating staining quality is crucial for enhancing diagnostic consistency and accuracy. This study was consequently undertaken to develop and validate a novel Endocytoscopy Staining Quality Assessment Scale. The primary objective was to overcome the current deficiency in standardized evaluation metrics by establishing a quantifiable approach to systematically assess and guide improvements in EC staining procedures. Ultimately, this scale aims to enhance operator proficiency and improve diagnostic precision in EC examinations.

## Methods

### Patients and image selection

This retrospective study included 334 patients who underwent colonoscopy and endocytoscopy at the Endoscopy Center of our institution between June 2023 and June 2024. All patients in this study underwent colonoscopy for a range of clinical indications, including routine screening, surveillance, or diagnostic assessment for symptoms such as abdominal pain, bloating, changes in bowel habits and rectal bleeding. Data were collected in two phases. From June to December 2023, a total of 2692 stained EC images were obtained from 202 patients for the purpose of evaluating the scientific validity and reliability of the assessment scale. From January to June 2024, 2413 additional images were collected from 172 patients to verify the applicability of the scale. All methods were carried out in accordance with regulations. The study protocol was approved by the Ethics Committee of the First Hospital of Jilin University (No. 2024 − 700). The requirement for informed consent was waived by the Ethics Committee of the First Hospital of Jilin University due to the retrospective nature of the study.

The same inclusion and exclusion criteria were applied consistently across both cohorts. EC images acquired from lesions assessed during diagnostic, screening, or surveillance colonoscopies. The exclusion criteria were: (1) absence of a corresponding histopathological diagnosis (2) incomplete essential clinical data and (3) duplicate images from the same lesion in a single patient. To minimize assessment bias, a rigorous dual-blinding procedure was implemented. All endoscopists evaluating images were blinded to patient identifiers, clinical outcomes, and the final histopathological diagnoses. Furthermore, images were presented in a randomized order to prevent recall bias.

To ensure representation of various lesion types, EC images were categorized into five groups: (1) normal mucosa; (2) benign lesions (including inflammatory polyps, juvenile polyps, hyperplastic polyps, and sessile serrated lesions without dysplasia); (3) adenomas (tubular adenomas < 10 mm with low-grade dysplasia and sessile serrated adenomas); (4) advanced adenomas (≥ 10 mm, presence of villous components, or high-grade dysplasia)^2,8^; and (5) adenocarcinomas (with varying degrees of differentiation).

For the development of this scoring system, we enrolled a large, consecutive image cohort (initial, *n* = 2692; validation, *n* = 2413). A post-hoc power analysis confirmed this sample size provides > 99.9% statistical power to detect a medium effect size (f = 0.25, α = 0.001) via an F-test, ensuring robust statistical validation.

### Endocytoscopy procedure

All endocytoscopy (EC) procedures included in this study were performed using the CF-H290ECI endocytoscope system (Olympus Co., Tokyo, Japan). This instrument provides an optical magnification capability of up to ×520, enabling visualization of a 570 × 500 μm tissue field of view. EC permits clear visualization of cellular nuclei and glandular lumina within the superficial epithelium at an approximate focal depth of 30 μm.In *vivo* staining was conducted using a 1% methylene blue (MB) solution as a single staining agent^2,8^. The stain was administered topically onto the surface of colorectal polyps and the adjacent normal mucosa via a non-traumatic spray catheter. Following a staining incubation period of approximately 1 min, excess stain was removed by gentle irrigation. Initial observation at lower magnification was performed to assess the mucosal pit pattern classification. Subsequently, the magnification was increased to the EC level (×520) for detailed examination and image acquisition of both the lesion and adjacent normal appearing mucosa. In instances where larger lesions exhibited heterogeneous histology (e.g., residual adenomatous components within an adenocarcinoma), the image selected for assessment was chosen to be most representative of the primary or most advanced pathological features identified. The entire assessment sequence, from irrigation to final image capture, was typically completed within an additional minute to ensure images were captured at peak staining quality.

Although methylene blue stains both the nucleus and cytoplasm, it is not a nucleus-specific dye. In this study, the scoring system evaluated nuclear delineation, defined as the observer’s ability to clearly discern cell morphology and nuclear contours under staining contrast.

### Endocytoscopy staining quality score scale

Staining quality was assessed based on four criteria, each scored on a 0–2 scale, yielding a total score ranging from 0 to 8: (1) nuclear clarity, (2) glandular lumen clarity, (3) surface mucus and dye rinsing degree, and (4) Image capture quality (including stabilization, focus, and brightness) (Table 1).

**Table 1:** Endocytoscopy staining quality score scale and scoring criteria.

Score	Nuclear clarity	Glandular lumen clarity	Surface mucus and dye rinsing degree	Image capture quality	Total score
0	< 50% area Nuclear clarity	< 50% area Glandular lumen clarity	< 50% area Complete mucus and dye wash-out	Poor stabilization: blurry image, poor focus and brightness, details not clearly discernible	
1	50%−70% area Nuclear clarity	50%−70% area Glandular lumen clarity	50%−70% area Complete mucus and dye wash-out	Average stabilization and focus, slightly dim brightness, partial clarity of details	
2	>70% area Nuclear clarity	>70% area Glandular lumen clarity	>70% area Complete mucus and dye wash-out	Good stabilization: Accurate focus, adequate brightness, clear details	

*Image capture quality includes: stabilization, focus, and brightness.

**Fig. 1:**
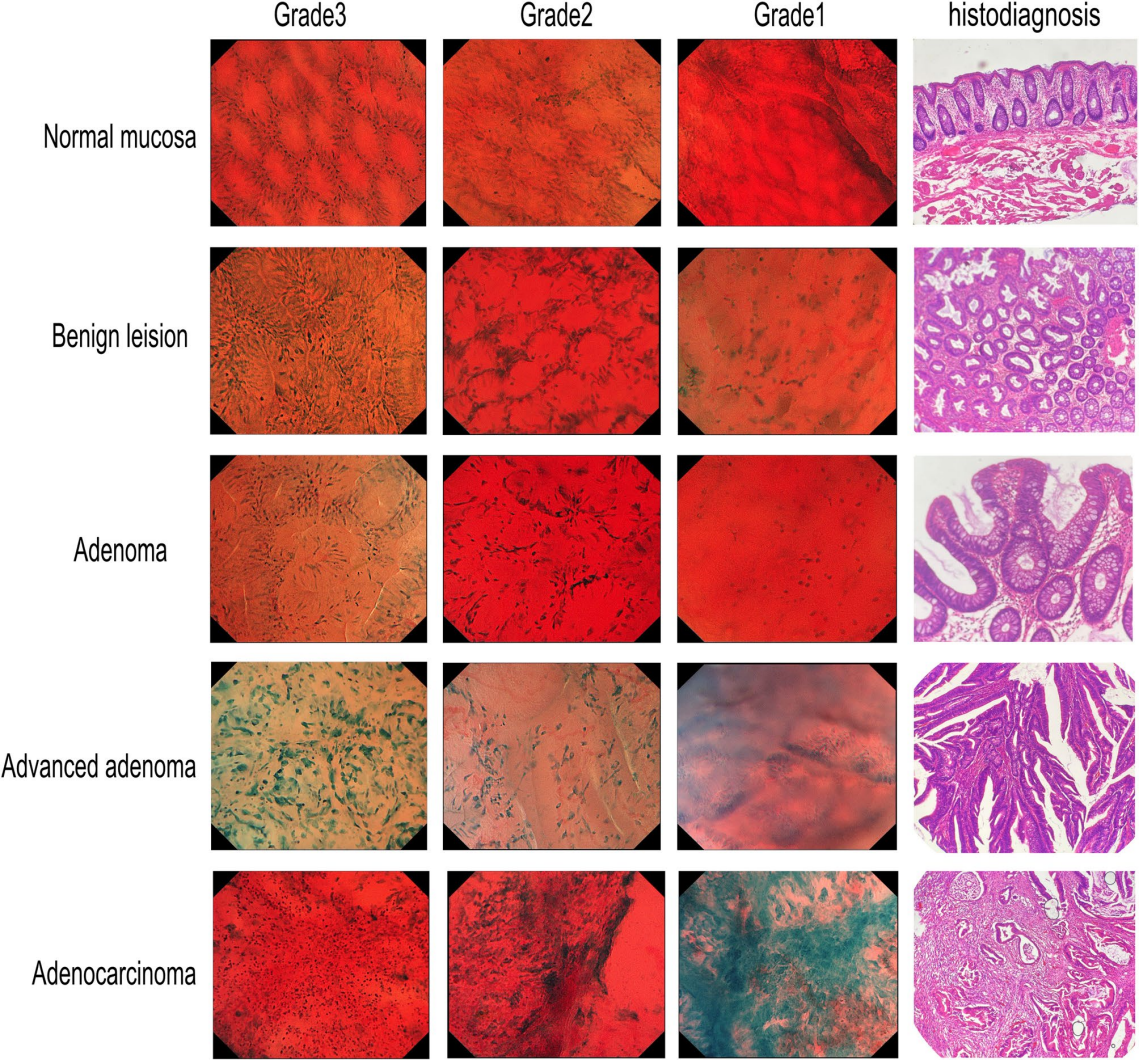
Representative images of endocytoscopy staining quality for different pathological types.

To objectively define staining grades, a K-Means cluster analysis was performed on the total scores. The optimal number of clusters (K = 3) was determined using both the Elbow method and Silhouette scores, confirming a natural three-group structure. This process partitioned the scores into three distinct groups ([0–2], [3–5], and [6–8]) with minimal overlap and significantly different mean scores (ANOVA, *P* < 0.001). Accordingly, we established these data-driven quality grades: Grade 1 (poor, ≤ 2 points), Grade 2 (fair, 3–5 points), and Grade 3 (high, ≥ 6 points). Representative EC images illustrating these grades are presented in Fig. 1.

### Definition and external validation of the gold standard score

Based on endoscopic expertise and learning proficiency, physicians were classified as either senior or junior endoscopists. Those who met all three of the following criteria were defined as senior: (1) more than 5 years of clinical experience, (2) over 200 endocytoscopy procedures performed, and (3) more than 3,000 total endoscopic examinations. Physicians who did not meet all criteria but had comparable experience were classified as junior.Three senior endoscopists independently evaluated the EC staining images in the initial cohort. In cases of scoring discrepancies, a consensus was reached through discussion. The final agreed-upon score was defined as the gold standard score.

**Fig. 2:**
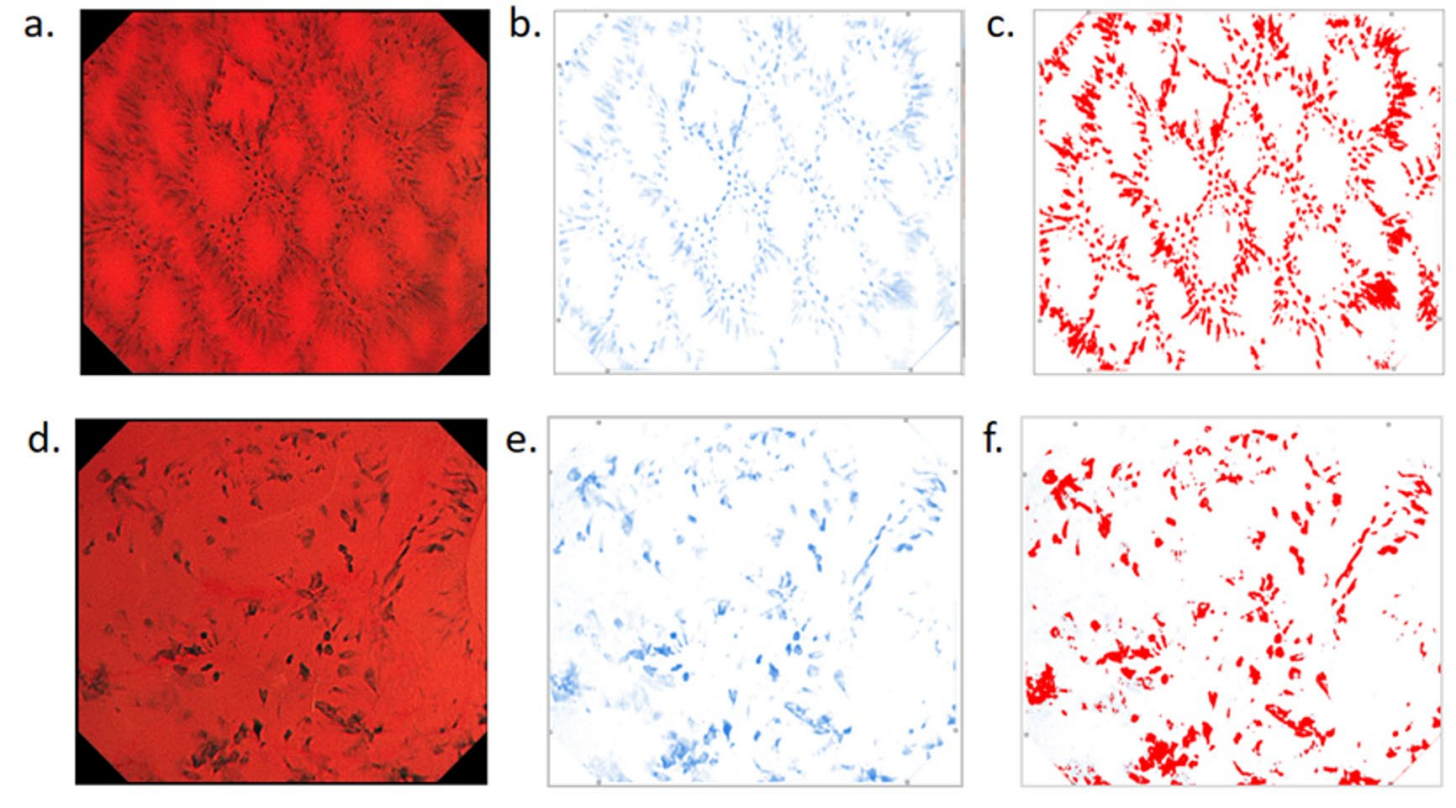
ImageJ analysis of endocytoscopy images. (**a**–**c**) Normal mucosa; (**d**–**f**) Adenoma. Panels (a) and (d) show endocytoscopic images after mucosal staining. Panels (b) and (e) demonstrate methylene blue extraction using ImageJ software. Panels (c) and (f) present area optical density (AOD) analysis of the selected regions.

To objectively assess the accuracy and reliability of the gold standard scores, external validation was performed. The Average Optical Density (AOD) of each EC image, representing staining intensity, was quantified using ImageJ software (Fig. 2). AOD values increase with deeper staining and can serve as an objective validation metric. However, in some cases, excessive residual dye on the mucosal surface resulted in falsely elevated AOD values, leading to potential false positives. To minimize this error, 291 images with excessive staining were excluded from the initial dataset. Spearman correlation analysis was then conducted on the remaining 2,401 images to validate the reliability of the gold standard scoring system.

### Training and validation of the score scale

To validate the scoring method, junior physicians independently evaluated the EC images from the initial cohort using the developed scale. Subsequently, a one-month standardized training program was conducted by senior physicians to unify scoring criteri. In the core practical component of the training, each junior physician was required to independently evaluate all 2,692 images from the initial cohort. During this process, senior physicians provided iterative feedback and focused guidance on discrepant scores to ensure a high level of consistency in the juniors’ understanding and application of the criteria across all four dimensions. After the training, junior physicians reassessed the EC images from the validation cohort. The improvement in inter-rater agreement before and after training was measured using Cohen’s kappa coefficient to evaluate the effectiveness of the training.

To assess the impact of staining quality on diagnostic accuracy, a team of senior physicians conducted blinded diagnoses of all EC images. The diagnostic outcomes were compared against histopathological results, which served as the reference standard, to determine diagnostic accuracy across different staining quality levels.

### Statistical analysis

According to the D’Agostino-Pearson omnibus test, baseline data for age were normally distributed and are presented as median (interquartile range, IQR); group differences were compared using one-way analysis of variance (ANOVA). Other categorical and ordinal variables, which were not normally distributed, are summarized as frequencies and percentages (n, %) and were compared between groups using the chi-squared (χ²) test. The reliability of the scale was assessed using Cronbach’s Alpha, and construct validity was assessed via exploratory factor analysis. Correlation analyses were performed using Spearman’s rank correlation. The results of the multinomial regression analysis are expressed as odds ratios (ORs) with their 95% confidence intervals (CIs). Inter-rater agreement among endoscopists was evaluated by calculating sensitivity, specificity, accuracy, Positive Predictive Value (PPV), Negative Predictive Value (NPV), and the Kappa coefficient.

All data analyses and image preparations were conducted using SPSS V.24.0 (IBM, USA), R V.4.2.1 (The R Foundation, Austria), and ImageJ V.1.54f (NIH, USA). *P* < 0.05 was considered statistically significant.

## Result

### Study flow diagram and patient demographics

The patient inclusion flowchart is detailed in Supplementary Fig. 1. A total of 2,692 EC images were included in the initial cohort, while 2,413 EC images were included in the validation cohort. Baseline patient characteristics are presented in Supplementary Table 1.

### Reliability and validity of the scale

The inter-rater reliability among three senior physicians for the evaluation EC staining images was 0.992. The internal consistency of the scale, as measured by Cronbach’s alpha, was 0.81. Among the individual components, image quality demonstrated the highest reliability (0.87), while the clarity of nuclear/glandular structures and mucus clearance showed moderate reliability (around 0.70) (Supplementary Fig. 2).Exploratory factor analysis (EFA) for validity testing revealed that the scale possesses a clear unidimensional structure, with the leading factor explaining 62.3% of the total variance. Except for the “image stabilization clarity” factor (loading = 0.453), which showed a slightly weaker association, the remaining three criteria were highly correlated with this factor (loadings > 0.83).

### External validation of the gold standard

**Fig. 3:**
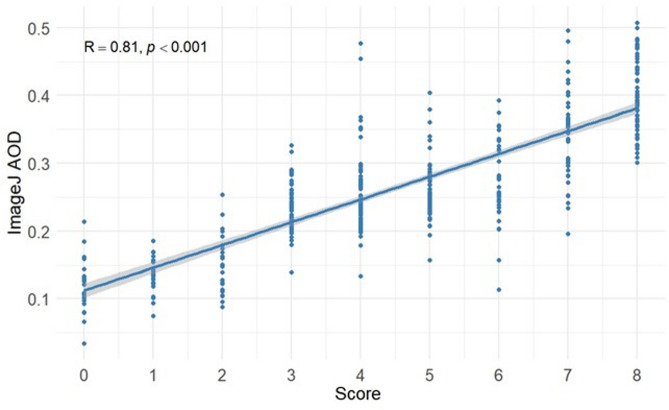
Correlation analysis between ImageJ AOD and the gold standard score. *The x-axis represents the gold standard score, and the y-axis represents the ImageJ Average Optical Density (AOD). Each point on the plot corresponds to the score and its associated AOD value for a single image. The solid diagonal line indicates the linear regression fit between the score and the AOD, illustrating the linear relationship between the two variables. The gray shaded area represents the 95% confidence interval for the regression line.

Spearman correlation analysis was performed between the AOD values of 2,401 EC stained images from the initial cohort and the total gold standard scores, as shown in Fig. 3. The results demonstrated a significant positive correlation, with ImageJ AOD values increasing as the scores increased (correlation coefficient *R* = 0.81, *P* < 0.001).

### Analysis of factors affecting image staining quality

**Fig. 4:**
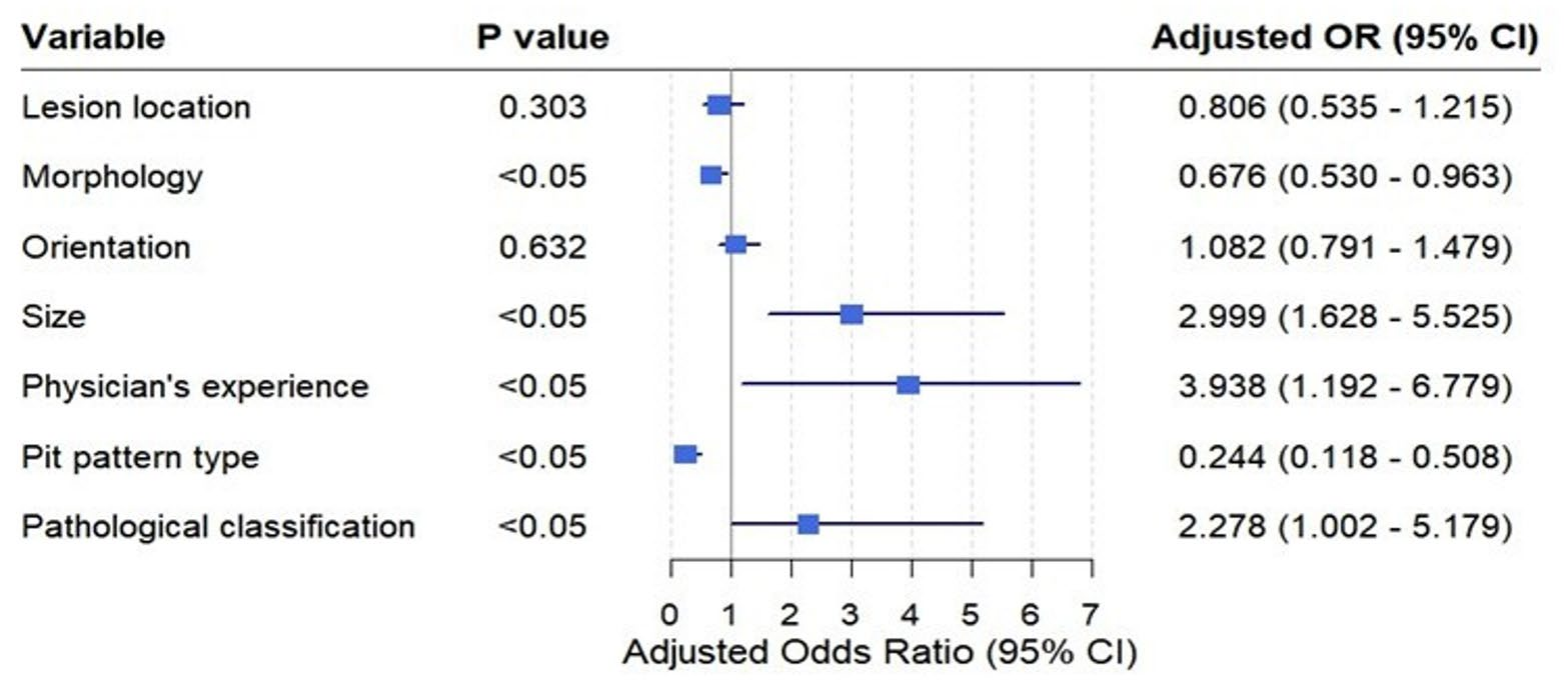
Multivariate logistic regression analysis of factors influencing staining quality.

To explore the key factors influencing image staining quality, we analyzed nine variables, including lesion location, orientation, morphology, size, pathological classification, pit pattern classification, physician experience, gender, and age. Chi-square testing (Table 2) revealed no significant differences in gender and age. Multinomial logistic regression analysis (Fig. 4) indicated that lesion morphology, lesion size, physician experience, pit pattern classification, and lesion characteristics were strong predictors of staining quality (*P* < 0.05). In contrast, lesion location and orientation showed no significant correlation with staining quality (*P* > 0.05).

**Table 2:** Analysis of factors affecting image staining quality.

	Grade1	Grade2	Grade3	Statistical value	*P* value
Lesion location, (n%)				χ²=20.68	<0.001
Distal	154 (27.4)	512 (38.5)	275 (34.2)		
Proximal	117 (20.9)	361 (27.2)	318 (39.5)		
Rectum	289 (51.6)	454 (34.2)	212 (26.3)		
Morphology, (n%)				χ²=31.14	<0.001
Normal mucosa	19 (3.3)	93 (7)	418 (51.9)		
0-Ip	31 (5.5)	66 (5.1)	10 (1.3)		
0-Isp	43 (7.7)	109 (8.2)	90 (11.2)		
0-Is	82 (15.4)	243 (18.3)	127 (15.8)		
0-IIa	180 (32)	439 (33.1)	113 (14)		
0-IIa + IIc	7 (1.2)	21 (1.6)	0 (0)		
LST	19 (3.3)	36 (2.7)	0 (0)		
Protrusion lesion	99 (17.6)	165 (12.4)	37 (4.6)		
Ulcerative lesion	80 (14.3)	155 (11.7)	10 (1.3)		
Lesion orientation during staining, (n%)				χ²=46.9	<0.001
Superior	122 (21.9)	284 (21.4)	106(13.1)		
Left	67 (12)	284 (21.4)	323 (40.1)		
Right	92 (16.4)	336 (25.3)	159 (19.7)		
Inferior	205 (36.6)	268 (20.2)	202 (25)		
Circumferential	74 (13.2)	155 (11.7)	15 (1.9)		
Size, median(n%)				χ²=47.6	<0.05
Normal mucosa	19 (3.3)	93 (7)	418 (51.9)		
<5 mm	153 (27.4)	177 (13.3)	31 (3.9)		
5–9 mm	129 (23.4)	495 (37.3)	123 (15.2)		
10–20 mm	110 (19.7)	253 (19.1)	202 (25)		
>20 mm	147 (26.3)	309 (23.3)	31 (3.9)		
Senior endoscopist, (n%)	110 (19.7)	893 (67.3)	678 (84.2)	χ²=105.14	<0.05
Pit pattern classification, median(n%)				χ²=139.1	<0.05
I	25 (4.4)	150 (11.3)	434 (53.9)		
II	166 (29.7)	294 (22.2)	111 (13.8)		
III	106(18.6)	377 (28.4)	185 (23)		
IV	99 (17.6)	207 (15.6)	49 (6.1)		
V	166 (29.7)	299 (22.6)	26 (3.2)		
Pathological classification, median(n%)				χ²=157.6	<0.05
Normal mucosa	18(3.3)	93 (7)	418 (51.9)		
Benign leision	172 (30.7)	268 (20.2)	105 (13.1)		
Adenoma	74 (13.2)	350 (26.4)	108 (13.4)		
Advanced adenoma	117 (20.9)	296 (22.3)	127 (15.8)		
Adenocarcinoma	179 (31.9)	320 (24.1)	47 (5.9)		
Male gender, (n%)	52.7%	53.1%	55.9%	χ²=6.51	0.11
Age(years), median(IQR)	51(45–59)	53 (43–63)	53(48–61)	F = 1.59	0.20

*Grade 1, Poor staining quality; Grade 2, Moderate staining quality; Grade 3, High staining quality IQR: Interquartile Range, LST: Laterally Spreading Tumor.

### External validation of the scale

The diagnostic performance of three junior endoscopists (A, B, and C) before and after training using the assessment scale is shown in Table 3. Prior to training, the kappa statistics for inter-rater agreement with the gold standard were 0.61, 0.66, and 0.69 for doctors A, B, and C, respectively. After one month of training, the kappa statistics for agreement with the gold standard improved to 0.81, 0.83, and 0.82, respectively.

**Table 3:** Diagnostic performance of junior endoscopists before and after scale-based training compared to the gold standard.

Grade 1	Endoscopist A	Trained Endoscopist A	Endoscopist B	Trained Endoscopist B	Endoscopist C	Trained Endoscopist C
Sensitivity, % (95% CI)	88.9 (81.7–93.9)	94.5 (87.6–98.2)	72.6 (63.6–80.5)	83.5 (74.3–90.5)	94.0 (88.1–97.6)	98.3 (94.0–99.8.0.8)
Specifcity, % (95% CI)	84.3 (80.3–87.8)	95.8 (93.4–97.5)	98.1 (96.2–99.2)	99.0 (97.5–99.7)	79.4 (74.9–83.3)	86.4 (82.6–89.7)
Accuracy, %(95% CI)	85.4 (81.9–88.4)	95.6 (93.4–97.2)	92.2 (89.5–94.3)	96.2 (94.1–97.7)	82.8 (79.2–86.0)	89.2 (86.1–91.8)
PPV, % (95% CI)	63.4 (55.5–70.8)	83.4 (74.9–90.1)	92.4 (84.9–96.9)	95.0 (87.7–98.6)	58.2 (50.8–65.3)	68.9 (61.2–75.8)
NPV, % (95%CI)	96.1 (93.4–97.9)	98.7 (97.1–99.5)	92.1 (89.1–94.5)	96.4 (94.2–98.6)	97.7 (95.4–99.1)	99.3 (97.8–99.9)
**Grade 2**						
Sensitivity, % (95% CI)	75.3 (69.8–80.7)	87.9 (83.3–91.6)	74.0 (67.9–79.5)	89.1 (84.6–92.6)	68.1 (61.7–73.9)	78.7 (73.0–83.8.0.8)
Specifcity, % (95% CI)	75.4 (69.8–80.5)	88.4 (83.8–92.2)	83.7 (78.7–88.0)	90.1 (85.6–93.5)	74.7 (69.0–79.8.0.8)	87.9 (83.4–91.6)
Accuracy, % (95% CI)	75.4 (71.4–79.1)	88.2 (85.0–90.9.0.9)	79.2 (75.3–82.7)	89.6 (86.6–92.1)	71.6 (67.4–75.5)	83.6 (80.1–86.7)
PPV, % (95% CI)	73.1 (67.1–78.6)	88.9 (84.5–92.5)	80.1 (74.2–85.3)	90.5 (86.2–93.8)	70.5 (64.1–76.3)	85.2 (79.8–89.7)
NPV, % (95%CI)	77.5 (71.9–82.5)	87.3 (82.6–91.3)	78.4 (73.2–83.1)	88.6 (84.0–92.3.0.3)	72.5 (66.8–77.7)	82.3 (77.4–86.6)
**Grade 3**						
Sensitivity, % (95% CI)	62.8 (54.5–70.6)	84.9 (78.2–90.1)	90.5 (84.6–94.7)	94.1 (89.1–97.3)	56.7 (48.4–64.9)	78.4 (70.9–84.7)
Specifcity, %(95% CI)	99.7 (98.4–99.9)	95.9 (93.3–97.8)	83.8 (79.5–87.5)	93.1 (89.9–95.5)	1 (98.9-1.9)	1 (98.9-1.9)
Accuracy, % (95% CI)	88.8 (85.7–91.4)	92.6 (89.9–94.7)	85.8 (82.4–88.7)	93.4 (90.9–95.4)	87.2 (83.9–90.0)	93.6 (91.1–95.6)
PPV, % (95% CI)	98.9 (94.2–99.9)	90.2 (84.1–94.5)	70.2 (63.1–76.5)	85.6 (79.4–90.5)	1 (95.7-1.7)	1 (96.8-1.8)
NPV, % (95%CI)	86.4 (82.7–89.6)	93.5 (90.4–95.8)	95.4(92.5–97.5)	97.2 (94.9–98.7)	84.6 (80.8–87.9)	91.6 (88.4–94.2)

* Grade 1, Poor staining quality; Grade 2, Moderate staining quality; Grade 3, High staining quality. PPV, positive predictive value; NPV, negative predictive value.

### Analysis of staining quality and EC diagnostic accuracy

The diagnostic accuracy of EC images in the groups with high, fair, and poor staining quality were 96.3%, 81.7%, and 45.1%, respectively (Table 4).

**Table 4:** Comparison between the diagnosis based on EC-stained images and histopathology.

Staining quality grouping	Total (*n*)	Total accuracy, %	Normal mucosa accuracy, %	Benign leision accuracy, %	Adenoma accuracy, %	Advanced adenoma accuracy, %	Adeno-carcinoma accuracy, %	*P* value (Compared with grade 3)
Grade 3	1526	96.3%	100%	97.4%	89.1%	91.4%	82.3%	-
Grade 2	2516	81.7%	86.2%	83.2%	83.8%	84.4%	72.4%	< 0.001
Grade 1	1063	45.1%	60%	57.8%	58.7%	48.6%	38.8%	< 0.001

## Discussion

The accuracy of endocytoscopic diagnosis largely depends on staining quality; however, standardized assessment criteria have been lacking. To address the key challenges in achieving high-quality staining and accurate diagnosis, we innovatively developed a four-dimensional endocytoscopic staining scale. Observing the morphology of the nucleus and glandular lumens is fundamental to the diagnostic utility of endocytoscopy^9^. For example, a Japanese study demonstrated that endocytoscopy can reliably differentiate between various types of serrated polyps by identifying characteristic morphological features such as the fusiform nuclei of TSA, the oval glandular lumens of SSA/P, and the star-shaped lumens of HP^10^. In the endocytoscopic evaluation of esophageal lesions, clear nuclear staining is especially critical. One study reported that 84% of esophageal squamous cell carcinoma cases could be diagnosed based on increased cell density and clear nuclear abnormalities, often eliminating the need for biopsy^6,11^.

In addition to the staining of target structures, other factors significantly affect the final diagnostic image quality. A study in Japan on human gastrointestinal tissue emphasized that, alongside optimizing staining with methylene blue and toluidine blue, complete mucus removalplays a decisive role in achieving high-quality images^8^. Moreover, endoscopist-induced image blurring due to hand tremors during image acquisition also decreases image quality and interferes with lesion assessment. Based on these diagnostic needs and in-depth discussions with the endoscopy team, we defined four key evaluation dimensions: nuclear clarity, glandular lumen clarity, surface mucus and dye rinsing degree, and image capture quality the key evaluation criteria. The high reliability and validity scores of the scale demonstrate its strong construct validity, making it a reliable diagnostic tool.

Multinomial regression analysis revealed that both lesion characteristics (morphology, size, pathology, and pit pattern classification) and physician experience significantly affect staining quality. For instance, tumor lesions can markedly influence staining outcomes. At the macroscopic level, features such as complex morphology, large lesion volume, and excessive mucus can result in spraying difficulties, inadequate dye penetration, or uneven staining. Microscopically, vascular and tissue alterations are also pivotal: fragile neovasculature prone to bleeding can obscure visualization and cause dye contamination, while stromal fibrosis and tissue densification limit dye diffusion^12^. Additionally, stromal fibrosis and tissue densification hinder dye penetration, while cancer cell dysplasia, disordered arrangement, and necrotic areas contribute to uneven and heterogeneous staining at the microscopic level^13^. Highly active lesions with specific patterns, such as Ip/Isp types, may also pose greater staining challenges due to gravitational effects.To optimize staining and imaging, several clinical strategies can be implemented: for tumor lesions, adjusting the staining duration, concentration, and spraying angle; thoroughly clearing mucus before staining and, if necessary, using mucolytics to enhance dye adhesion and penetration; adjusting patient positioning to improve staining uniformity; and enhancing physician training with standardized procedures.

Our findings demonstrate that the EC staining scale holds broad clinical applicability, particularly in enhancing endoscopists’ staining skills and ensuring diagnostic accuracy. First, the scale provides an objective evaluation of physicians’ staining proficiency and serves as a guide for training, helping them identify and improve weaknesses, thereby enhancing their skill level. Second, the study confirmed that high-quality staining is a key factor for the diagnostic accuracy of EC images. By controlling the quality, the scale helps prevent misdiagnosis and missed diagnoses caused by image-related issues. Moreover, the scale provides a standardized benchmark for team discussions and quality control, fostering standardized communication, reducing discrepancies due to experience-related biases, and improving overall team diagnostic and treatment quality.

The innovation of this study lies in developing and validating the first multidimensional scoring system for evaluating endocytoscopic staining quality, addressing the long-standing challenge of standardized assessment. This framework provides a key prerequisite for future artificial intelligence–based image analysis and quality control, paving the way for the development of intelligent tools capable of real-time image quality optimization and diagnostic assistance. Although the present study demonstrated the scoring system’s potential value in improving immediate clinical decision-making, its translation into long-term patient outcomes remains to be verified. Therefore, prospective studies are urgently needed to investigate its association with critical clinical endpoints such as lesion recurrence, survival, and optimization of surveillance strategies. While this score has been validated for colonic polyp detection, extending its evaluation to oesophageal, gastric, and small bowel EC will be important to confirm generalizability. In addition, incorporating EC-based assessment of mucosal healing and barrier integrity in IBD represents a promising future application^14,15^.

This study has several limitations. First, the data were obtained from a single medical center; therefore, large-scale, multicenter studies are warranted to validate the generalizability and robustness of the proposed scoring system across diverse clinical settings and patient populations. In addition, it should be acknowledged that confocal endomicroscopy is not yet widely implemented worldwide, and thus, the value of this study lies primarily in its methodological and educational contribution.

## Conclusion

In conclusion, the endocytoscopic staining quality score scale developed in this study provides clinicians with a reliable and validated tool to improve examination quality and diagnostic precision. Through a rigorous methodological design and validation process, we demonstrated its strong applicability and effectiveness in clinical practice.

## Supplementary Information

Below is the link to the electronic supplementary material.

Supplementary Material 1

## Data Availability

Data available on request from the corresponding author.
