# Supplementary material for: Development and validation of a novel scoring scale for colonic endocytoscopy staining quality
Source: Sci Rep. 2026 Feb 4;16:7301. doi: 10.1038/s41598-026-37406-0 (PMC12923546; doi:10.1038/s41598-026-37406-0)
Supplement: Supplementary file 1 — Supplementary Material 1 [file 41598_2026_37406_MOESM1_ESM.docx]

**Supplementary figure 1** Flowchart of patient enrollment for the (a) initial cohorts and (b) validation cohorts.


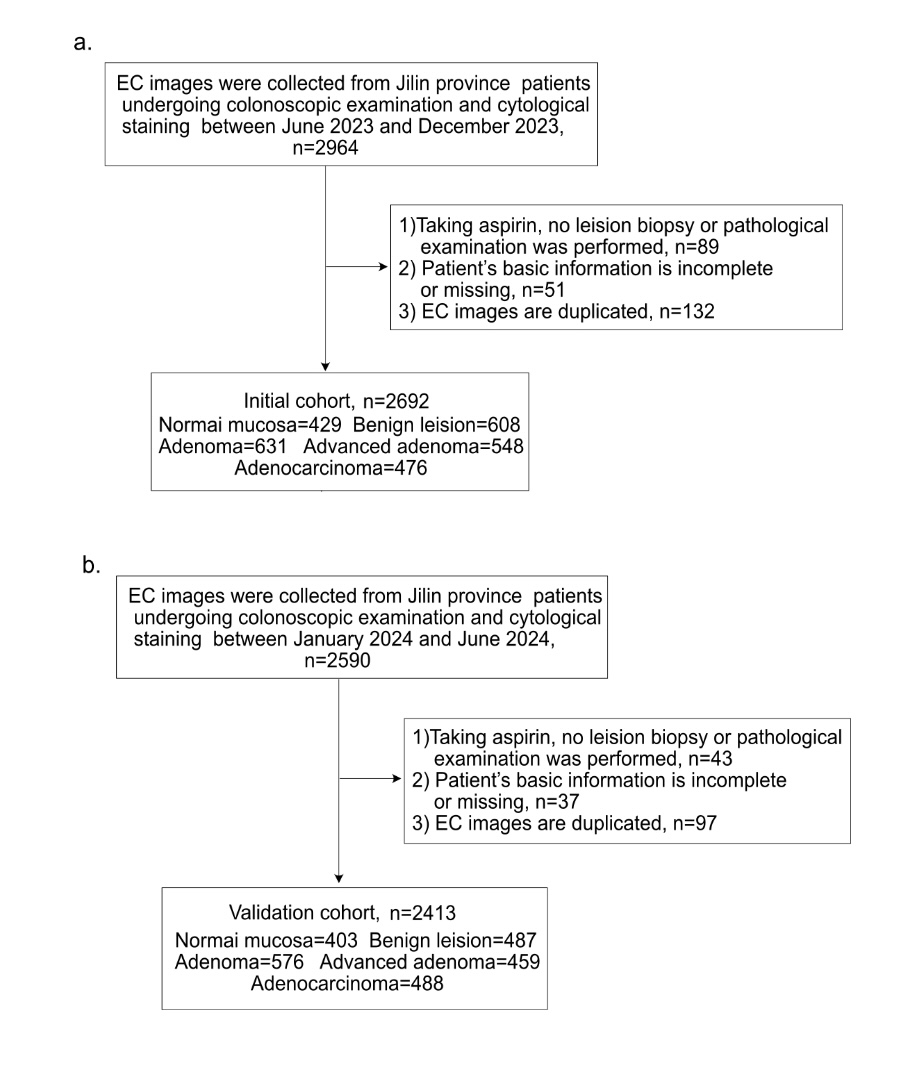


**Supplementary figure 2** Reliability analysis plot. Reliability analysis of the scale.


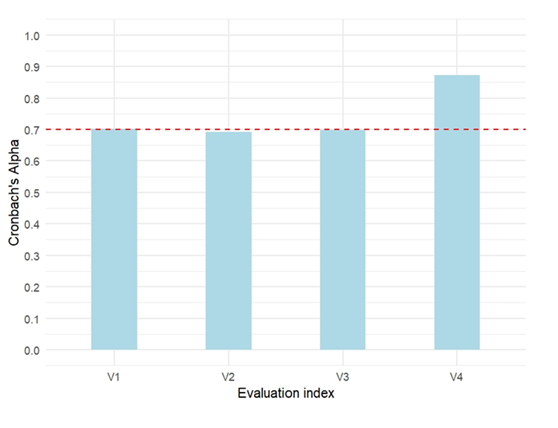


This plot displays the Cronbach's Alpha values for the four assessment dimensions: V1 (Clarity of nuclei) = 0.70, V2 (Clarity of glandular lumens) = 0.69, V3 (Mucus and dye clearance) = 0.70, and V4 (Image acquisition quality) = 0.87. All values are close to or exceed the minimum acceptable standard of 0.7, which is indicated by the red dashed line.

**Supplementary table 1** Basic demographics of initial cohor and validation cohorts.

|  | Initial cohort (n=2692) | | | | | | Validation cohort (n=2413) | | | | | |  |
| --- | --- | --- | --- | --- | --- | --- | --- | --- | --- | --- | --- | --- | --- |
| Characteristic | Normal mucosa | Benign leision | Adenoma | Advanced adenoma | Adenocarcinoma | P value  (within-group) | Normal mucosa | Benign leision | Adenoma | Advanced adenoma | Adenocarcinoma | P value  (within-group) | P value  (Between-group) |
| Male gender,(n%) | 366 (13.6) | 366 (13.6) | 323 (12) | 382 (14.2) | 350 (13) | 0.54 | 319 (13.2) | 290(12) | 338 (14) | 290 (12) | 314 (13) | 0.53 | 0.47 |
| Age(years),  median(IQR) | 52.4  (49.3-56) | 50.5  (43-57) | 51.6  (45-55.8) | 53.2  (45-59.8) | 54.2  (52-58) | 0.1 | 52.9  (46-61) | 53.0  (45-58.5) | 53.2  (46.3-63.5) | 53.8  (45.7-63) | 54.2  (46-62.8) | 0.89 | 0.11 |
| Size, (n%) | ＜0.05 | | | | | | ＜0.05 | | | | | | 0.13 |
| ＜5mm | - | 216 (8) | 199 (7.4) | 0 | 0 |  | - | 217 (9) | 142 (5.3) | 24 (1) | 0 |  |  |
| 5-9mm | - | 245 (9.1) | 317 (11.8) | 120 (4.5) | 39 (1.4) |  | - | 241 (10) | 314 (13) | 96 (4) | 24 (1) |  |  |
| 10-20mm | - | 147 (5.4) | 115 (4.3) | 256 (9.5) | 217 (8.1) |  | - | 29 (1.2) | 120 (5) | 214 (8.9) | 78 (3.2) |  |  |
| ＞20mm | - | 0 | 0 | 172 (6.4) | 220 (8.2) |  | - | 0 | 0 | 125 (5.2) | 386 (16) |  |  |
| Lesion location, (n%) | ＜0.05 | | | | | | ＜0.05 | | | | | | 0.5 |
| Distal | 128 (4.7) | 264 (7.2) | 237 (8.8) | 164 (6.1) | 79 (2.9) |  | 70 (2.9) | 199 (8.2) | 240 (10) | 168 (7) | 96 (4) |  |  |
| Proximal | 165 (6.1) | 183 (6.8) | 201  (7.4) | 231 (8.6) | 142 (5.3) |  | 261 (10.8) | 144 (6) | 140 (5.8) | 150 (6.2) | 48 (2) |  |  |
| Rectum | 136 (5.1) | 140 (5.2) | 156 (5.8) | 140 (5.2) | 255 (9.5) |  | 72 (3) | 144 (6) | 138 (5.7) | 117 (4.8) | 301 (12.5) |  |  |
| Mutilocation | 0 | 21 (0.8) | 37 (1.4) | 13 (0.5) | 0 |  | 0 | 0 | 58 (2.4) | 24 (1) | 43 (1.8) |  |  |
| Total number of intestinal lesions, (n%) | 0.12 | | | | | | ＜0.05 | | | | | | 0.19 |
| 1-2 | - | 436 (16.2) | 361 (13.4) | 307 (11.4) | 245 (9.1) |  | - | 288 (12) | 384 (16) | 216 (9) | 408 (17) |  |  |
| 3-5 | - | 172 (6.4) | 227 (8.4) | 129 (4.8) | 188 (7) |  | - | 168 (7) | 192 (7.9) | 144 (6) | 48 (2) |  |  |
| ＞5 | - | 0 | 43 (1.6) | 112 (4.2) | 43 (1.6) |  | - | 31 (1.3) | 0 | 99 (4.1) | 32 (1.3) |  |  |

***** Distal colon: ascending colon and transverse colon, Proximal colon: descending colon and sigmoid colon, Multiple sites: lesions involving two or more anatomical locations
